# Pembrolizumab in vaginal and vulvar squamous cell carcinoma: a case series from a phase II basket trial

**DOI:** 10.1038/s41598-021-83317-7

**Published:** 2021-02-11

**Authors:** Jeffrey A. How, Amir A. Jazaeri, Pamela T. Soliman, Nicole D. Fleming, Jing Gong, Sarina A. Piha-Paul, Filip Janku, Bettzy Stephen, Aung Naing

**Affiliations:** 1 Department of Gynecologic Oncology and Reproductive Medicine, The University of Texas MD Anderson Cancer Center, Houston, TX USA; 2 Division of Cancer Medicine, Department of Investigational Cancer Therapeutics, The University of Texas MD Anderson Cancer Center, 1400 Holcombe Blvd, FC8.2026, Houston, TX 77030 USA

**Keywords:** Gynaecological cancer, Cancer immunotherapy

## Abstract

Vaginal and vulvar squamous cell carcinoma (SCC) are rare tumors that can be challenging to treat in the recurrent or metastatic setting. We present a case series of patients with vaginal or vulvar SCC who were treated with single-agent pembrolizumab as part of a phase II basket clinical trial to evaluate efficacy and safety. Two cases of recurrent and metastatic vaginal SCC, with multiple prior lines of systemic chemotherapy and radiation, received pembrolizumab. One patient had significant reduction (81%) in target tumor lesions prior to treatment discontinuation at cycle 10 following confirmed progression of disease with new metastatic lesions (stable disease by irRECIST criteria). In contrast, the other patient with vaginal SCC discontinued treatment after cycle 3 due to disease progression. Both patients had PD-L1 positive vaginal tumors and tolerated treatment well. One case of recurrent vulvar SCC with multiple surgical resections and prior progression on systemic carboplatin had a 30% reduction in her target tumor lesions following pembrolizumab treatment with a PD-L1 positive tumor. Treatment was discontinued for grade 3 mucositis after cycle 5. Pembrolizumab may provide some clinical benefit to some patients with vaginal or vulvar SCC and is overall safe to utilize in this population. Future studies are needed to evaluate the efficacy of pembrolizumab in these rare tumor types and to identify predictive biomarkers of response.

## Introduction

Vaginal and vulvar cancers are rare malignancies that have similar estimated incidence (0.7 and 2.6 diagnoses per 100,000 women per year, respectively) and mortality (0.2 and 0.6 deaths per 100,000 women per year, respectively) rates in the United States^1^. Due to the difficulty of performing large prospective randomized trials in these rare tumor populations, systemic chemotherapeutic regimens have generally been extrapolated from experience in the management of cervical cancer as these malignancies share similar epidemiologic risk factors, are predominantly of squamous cell carcinoma (SCC) histologic subtype, and are strongly associated with human papilloma virus (HPV) infection^2–4^. However, vaginal and vulvar cancers can be challenging to treat when disease is not amenable to radiation or surgical resection^5,6^. Treatment response rates to systemic chemotherapeutic regimens are variable in the recurrent setting for vaginal and vulvar SCC; there is currently no consensus on effective regimens^5–8^. Additionally, given the propensity of vaginal and vulvar SCC to develop at older stages of life, treatment options may be further limited by associated toxicity and morbidity^3,4^.

There has been a growing interest for immunotherapy in the field of oncology. Immune checkpoint inhibitors have demonstrated impressive, durable responses even among patients who have undergone multiple lines of prior systemic therapy. Specifically, use of immunotherapy in HPV-related cancers is of particular interest given that carcinogenesis is associated with the inability of the immune system to clear the virus^9,10^. An anti-PD-1 monoclonal antibody, pembrolizumab blocks the PD-1/PD-L1 pathway (an escape mechanism that malignant cells use to evade immune surveillance) thereby augmenting T-cell mediated anti-tumor activity.

Pembrolizumab has demonstrated impressive clinical response in microsatellite instability high (MSI-H) or mismatch repair deficient (dMMR) tumors, which led to the US Food and Drug Administration (FDA) approval for use in MSI-H/dMMR solid tumors^11,12^. In gynecologic cancers, single-agent pembrolizumab has FDA approval for MSI-H endometrial cancer (response rates 53–57.1%) or PD-L1 positive (Combined Positive Score ≥ 1%) cervical cancer (response rates 12.2–17%) who have progressed on prior systemic therapy^11–14^. As the first drug to receive FDA approval for a tissue agnostic indication, pembrolizumab may be of clinical benefit in other solid tumors and possibly among those with HPV-associated gynecologic cancers such as vaginal or vulvar SCC. Given the rarity of vaginal or vulvar SCC, few studies have examined the use of immune checkpoint inhibitors in these tumor types.

Thus as part of a clinical phase II basket trial for patients with advanced rare malignancies, we report three cases of SCC (two vaginal and one vulvar) who were treated with single-agent pembrolizumab.

## Cases

### Clinical trial design

We present three patients with recurrent, metastatic vaginal (patient 1 and 2) or vulvar (patient 3) SCC who were enrolled into cohort 10 (“other rare tumor histologies” category) of an open-label, phase II basket clinical trial (ClinicalTrials.gov: NCT02721732) at the University of Texas MD Anderson Cancer Center (enrolment period August 15, 2016–July 27, 2018). In brief, the trial sought to examine the clinical efficacy and safety of single-agent pembrolizumab (200 mg IV every 3 weeks) in 10 prespecified cohorts of advanced, rare tumors regardless of PD-L1 status. Trial design and overall trial results are described elsewhere^15^. All trial patients had PD-L1 and tumor infiltrating lymphocyte (TIL) characterization of tumor tissue that was correlated with treatment response, as described previously^15^. PD-L1 characterization was reported as Combined Positive Score (CPS) which is defined as the number of PD-L1 staining cells divided by the total number of viable cells, multiplied by 100. CPS ≥ 1% denoted PD-L1 positive expression^16^. Treatment response was evaluated using Immune-related Response Evaluation Criteria in Solid Tumors (irRECIST) guidelines on serial radiologic imaging at baseline, every 9 weeks until the first 6 months followed by every 12 weeks at the discretion of the investigator. Safety and tolerability were assessed by characterization and grading of adverse events via the National Cancer Institute Common Terminology Criteria for Adverse Events (CTCAE) v4.03. All patients provided informed consent prior to enrolment. Furthermore, the protocol was approved by the FDA and the Institutional Review Board at The University of Texas MD Anderson Cancer Center. The study was conducted in accordance with the Declaration of Helsinki and the International Conference on Harmonization Good Clinical Practice guidelines.

### Vaginal SCC cases

Baseline clinical and tumor characteristics as well as prior treatment histories are demonstrated in Tables 1 and 2, respectively. The two patients (patient 1 and 2) were 72 and 63 years old (respectively) women with recurrent vaginal cancer and had grade 2 SCC histologies with infra- and supra-diaphragmatic metastatic disease. Both patients had multiple lines of prior systemic therapy (four and six, respectively) and prior radiation therapy before trial enrolment. Among prior systemic therapies received, the treatment with the longest duration of disease control after first disease recurrence for patient 1 was carboplatin/paclitaxel/bevacizumab followed by maintenance bevacizumab (7 months total). For patient 2, the systemic treatment with the longest duration of disease control following first recurrence was selinexor/eribulin (6 months).

**Table 1:** Baseline clinical and tumor characteristics.

Pt	Age	Cancer	Grade	ECOG PS	Years since initial diagnosis	FIGO 2009^a^	Sites of metastasis prior to treatment^b^	PD-L1 CPS	TIL infiltration
1	72	Vaginal	2	1	3	IVB	AA, AN, AT, N	5	2
2	63	Vaginal	2	1	3	IVB	AA, B, H, L,	2	2
3	88	Vulvar	1	1	20	I	AR, V	5	2

CPS = Combined Positive Score, defined as the number of PD-L1 staining cells divided by the total number of viable cells, multiplied by 100; CPS ≥ 1% denoted PD-L1 positive expression ECOG PS = Eastern Cooperative Oncology Group Performance Status. N/A = Not applicable. Pt = patient. TIL infiltration = intensity of tumor-infiltrating lymphocytes within tumor nests on a scale of 0 to 3; 0 = absence of TIL, 1 = few amounts of TIL, 2 = moderate amounts of TIL, 3 = high amount of TIL. SCC = squamous cell carcinoma.

^a^International Federation of Gynecology and Obstetrics (FIGO) 2009 staging at diagnosis.

^b^Sites of metastatic disease prior to treatment: AA = adenopathy of the abdomen, AN = adenopathy of the neck, AT = adenopathy of the thorax, AR = anorectum, B = bone, H = hepatic metastases, L = lung metastases, N = neck, V = vagina.

**Table 2:** Prior treatments before pembrolizumab treatment.

Pt	Prior radiation treatments	Number of prior surgeries	Number of prior systemic therapies	Prior systemic therapies^a^
1	1. External beam radiation therapy and brachytherapy 2. Chemoradiation^b^	0	4	1. Carboplatin/paclitaxel 2. Cisplatin^b^ 3. Carboplatin/paclitaxel/bevacizumab followed by maintenance bevacizumab^c^ 4. Topotecan/bevacizumab
2	1. Palliative radiation to the groin and neck	0	6	1. Carboplatin/paclitaxel 2. Cisplatin/topotecan 3. Cetuximab/prexasertib 4. Sapanisertib/afibercept 5. Bimiralisib 6. Selinexor/eribulin^c^
3	1. External beam radiation therapy	11	1	1. Carboplatin^c^

Pt: patient.

^a^Lines of systemic therapy are ordered chronologically.

^b^Radiation was given concurrently with weekly cisplatin.

^c^Longest duration of disease control on a systemic therapeutic regimen following recurrence.

**Figure 1:**
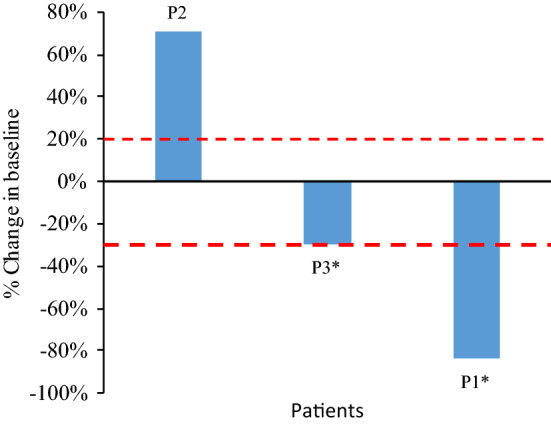
Radiologic response to pembrolizumab in patients with vaginal or vulvar cancer. Waterfall plot illustrating the best objective response to pembrolizumab in three evaluable patients using irRECIST criteria. Each bar represents a patient and shows the maximum percentage change from baseline in the sum of the longest diameters of all target lesions and any new lesions while on pembrolizumab. The area above the upper red dotted line represents progressive disease (≥ 20% increase in the sum of the diameters of the target lesions compared with the baseline). The area between both upper and lower red dotted lines represents stable disease. The area below the lower red dotted line represents treatment response (≥ 30% increase in the sum of the diameters of the target lesions compared with the baseline). P1–3: Patient 1–3. *Patient 1 and 3 had unconfirmed partial responses as defined by irRECIST criteria and would be classified as having stable disease as the best objective response.

**Figure 2:**
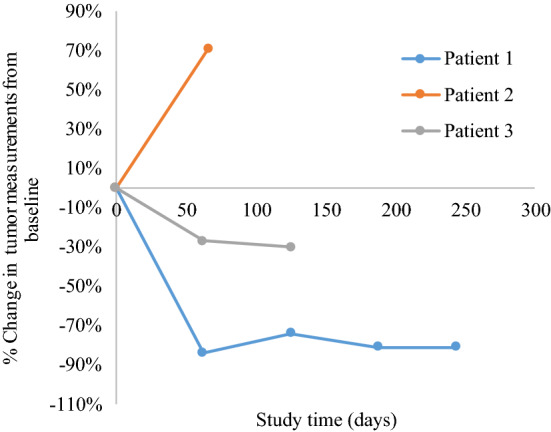
Tumor response by irRECIST across time. This spider plot demonstrates the tumor measurements from baseline using irRECIST criteria during the course of treatment with pembrolizumab in the three patients. Patient 1 and 2 had progression of disease prior to stopping treatment while patient 3 stopped treatment due to treatment-related toxicity. Patient 1 and 3 had unconfirmed partial responses as defined by irRECIST criteria. Despite a significant reduction in the size of the target lesions on her initial scan at cycle 3 with partial response, her follow-up scans at cycle 6 and cycle 9 demonstrated the appearance of new lesions and progression of non-measurable lesions; the best objective response is stable disease. Patient 3 had an unconfirmed partial response due to treatment discontinuation (after cycle 5) following initial evaluation of partial response; the best objective response is stable disease.

Figures 1 and 2 demonstrate the best percentage change in tumor target lesions compared to baseline and the dynamic changes in tumor measurements compared to baseline, respectively. With single-agent pembrolizumab, patient 1 had a 68% decrease in the size of the target lesions following three cycles and this continued with increasing cycles of treatment (74% decrease after cycle 6 and 81% decrease after cycle 9). Despite both progressive reduction of the size of the target lesions and clinical benefit, patient 1 had developed new nodal lesions and progression of non-measurable lesions after cycle 6 and this was confirmed on subsequent imaging studies. Per trial protocol, the patient was permitted to continue pembrolizumab following confirmation of disease progression until repeat radiologic imaging 4 weeks after the confirmatory scan as long as she was clinically stable. Repeat radiologic imaging demonstrated disease progression and thus a total of 10 cycles was administered prior to treatment discontinuation. Patient 1’s best objective response was classified as stable disease using irRECIST criteria. For patient 2, there was no objective response. There was a 71% increase in the patient’s tumor measurements according to irRECIST criteria following three cycles of pembrolizumab and treatment was discontinued. Her best objective response was classified as progressive disease (Figs. 1 and 2).

Overall, the treatment was tolerable for both patients with grade 2 fatigue as the only treatment-related adverse event (TRAE) for patient 2 and this was observed after cycle 3. Patient 1 did not have any TRAEs.

For tumor characterization, PD-L1 expression was positive in both patients with a CPS score of 5 and 2, respectively. Both patients’ tumors had moderate amounts of TIL (score 2 on a scale of 0 to 3).

### Vulvar SCC case

Patient 3 was an 88-year-old woman with a grade 1 SCC and long-standing disease with the bulk of the suspected disease recurrence involving the vagina followed by the ano-rectal region (Table 1). This patient with vulvar cancer had multiple surgical interventions (eight cytoreductive and three ablative procedures), radiation therapy, and most recently had disease progression on single-agent carboplatin prior to study enrolment (Table 2). Although her disease localization made a pelvic exenteration a possible treatment option, there was consensus between the patient, her medical provider, and a multidisciplinary tumor board that the procedure could result in excessive surgical morbidity (given her age). Thus, the patient opted for clinical trial enrolment and was ultimately included into the vaginal cancer expansion cohort of the phase II trial with pembrolizumab for several reasons. First, there was no available cohort of vulvar SCC on trial given the rarity of the tumor. Second, as HPV-related malignancies, systemic treatment of vaginal and vulvar cancers are very similar with strategies extrapolated from cervical cancer studies. Finally, it was difficult to determine with absolute certainty that her disease would be related to recurrent vulvar cancer rather than de novo vaginal cancer given her vaginal biopsies also demonstrated SCC. Due to the bulk of her disease was in the vagina, the patient was treated in the vaginal cancer expansion cohort of trial.

On pembrolizumab, there was a 27% reduction in the target lesions following three cycles and a 30% reduction following another two cycles (Figs. 1 and 2). Due to a grade 3 TRAE, treatment was discontinued after cycle 5 before a confirmatory scan for partial response could be performed. Thus, her best objective response was stable disease by irRECIST criteria. Patient 3 reported a grade 1 maculopapular rash following cycle 1 and grade 2 pruritus after cycle 4. Grade 3 mucositis led to treatment discontinuation. All the aforementioned TRAEs were immune-related. For tumor characterization, patient 3 had positive PD-L1 expression (CPS score of 5) and moderate amounts of TIL (score 2 on a scale of 0 to 3).

### Disclaimer

The views expressed in this manuscript are the authors’ own views and are not the official position of the institution or supporting funding sources.

### Ethics approval and consent to participate

The protocol was approved by the US Food and Drug Administration (FDA) and the Institutional Review Board at the University of Texas MD Anderson Cancer Center. The study was conducted in accordance with the Declaration of Helsinki and the International Conference on Harmonization Good Clinical Practice guidelines. All the study participants provided written informed consent before enrollment.

### Consent for publication

All the study participants provided written informed consent prior clinical trial enrollment.

## Discussion

In this case series, we present two patients (patient 1 and 2) with recurrent vaginal SCC and one patient (patient 3) with recurrent vulvar SCC who received single-agent pembrolizumab as part of a phase II basket trial. Although there were no confirmed objective responses that were observed, patient 1 had a significant reduction in size of the tumor measurements on radiologic imaging (61% decrease) following cycle 3 of pembrolizumab. However, this objective response was not confirmed on repeat imaging after cycle 6. In her case, subsequent imaging showed new metastatic lesions with progression of non-target lesions despite persistent reduction in the measured target lesion sizes (74% followed by 81% decrease). Patient 3 had a 30% reduction in target lesions after five cycles of pembrolizumab but discontinued study treatment due to a grade 3 TRAE (oral mucositis) before repeat imaging could confirm a partial response. Thus, it is possible that the treatment could have resulted in further tumor reduction. In contrast, patient 2 had no tumor reduction and had significant disease progression after 3 cycles of pembrolizumab.

Multiple factors may explain differences in clinical benefit to pembrolizumab for these patients. The first attributable factor may be the difference PD-L1 expression in the tumor microenvironment as evidenced by the PD-L1 CPS scores of 5, 2, and 5 for patients 1–3, respectively. PD-L1 status has been described as a predictive biomarker of response to PD-1 inhibitor monotherapy and increasing PD-L1 expression has been associated with improved response^16–19^. Thus, the higher expression of PD-L1 in patient 1 and 3 (CPS score 5) may explain a better response compared to patient 2 (CPS score 2) despite similar amounts of TIL (another predictive biomarker for response)^17–19^. Immunosuppressive factors in the tumor microenvironment may also modulate response to PD-1 inhibitors despite PD-L1 expression (CPS ≥ 1%) and TIL infiltration, leading to poor response in patient 2. Unfortunately, evaluation of possible contributory immunosuppressive factors was not part of the trial design. Furthermore, pembrolizumab is observed to respond more favorably in tumors with high tumor mutational burden or microsatellite instability-high status. For trial participation, patients were not required to have testing for these aforementioned molecular features and it is possible that their tumors could have had low mutational burden or been microsatellite stable.

Although there was not a confirmed partial response for patient 3, there was a trend for tumor reduction. This tumor reduction may have persisted if treatment was not discontinued due to a grade 3 toxicity (oral mucositis). This association between immune-related adverse events and relatively improved treatment response is suggestive of an active immune system and this observation has been similarly reported in multiple studies^20–22^.

Due to tumor rarity and lack of strong trial data, management of recurrent/metastatic vaginal and vulvar SCCs has largely been extrapolated from treatment strategies in cervical cancer. These strategies have typically consisted of platinum-based systemic combinations when chemoradiation or surgery is not possible^5,6^. These systemic combinations have not been consistently efficacious in these patient populations. In vaginal cancer, efficacy of systemic treatment has been largely anecdotal but one study has demonstrated an ORR of 6.25% for single agent cisplatin in 16 patients^5,7^. For vulvar cancer, studies evaluating systemic chemotherapy regimens have had small sample sizes with variable response rates (0–40%)^6,23^. Currently there are no standardized systemic regimens for vaginal and vulvar cancers^5,6,23^. Thus, other alternative treatment therapies have been investigated, with immunotherapy gaining interest. In this present study, two of the three patients with vaginal or vulvar SCC were observed to derive clinical benefit (stable disease as per irRECIST criteria). Two other studies have demonstrated some clinical benefit for these tumor types. In a phase I/II trial (CheckMate 358), nivolumab (PD-1 inhibitor) monotherapy efficacy was evaluated in a small cohort of recurrent/metastatic vaginal or vulvar SCC^24^. Although a small sample size (two vaginal and three vulvar cancers), the investigators observed a 20% objective response rate (one partial response in a vulvar cancer patient) with 40% of patients with 6 months of disease control^24^. In another case report of vulvar SCC, the patient with a PD-L1 positive tumor was observed to have a complete response to pembrolizumab^25^. In CheckMate 358 and the aforementioned case report, PD-1 inhibitor therapy was well-tolerated with no significant TRAEs^24,25^.

Strengths of our case series include the evaluation of the use of pembrolizumab in a rare cohort of vaginal and vulvar SCC with correlation to translational PD-L1 and TIL data. It represents the first reported use of pembrolizumab for vaginal SCC. Furthermore, tumor responses were objectively measured using irRECIST criteria. As mentioned earlier, tumor mutational burden characterization and microsatellite-instability status testing was not a requirement for patients who were included in this phase II clinical trial and this represents a study limitation.

In conclusion, single-agent pembrolizumab was shown, in this study, to be generally safe to utilize for vaginal and vulvar SCC and has demonstrated some clinical benefit. However, given the small size of this cohort of patients, future studies should examine the role of pembrolizumab in the treatment of vaginal and vulvar SCC. Predictive biomarkers (possibly established in other SCC cohorts such as cervical, anal, or head and neck cancers) should be investigated to identify patients who would derive the greatest clinical benefit.

## Data Availability

Data are available upon reasonable request. The datasets used and/or analyzed during the current study are available from the corresponding author on reasonable request and approval from study sponsor according to available guidelines at time of request.

## References

[1] Siegel RL, Miller KD, Jemal A (2020). Cancer statistics, 2020. CA Cancer J. Clin..

[2] Daling JR (2002). A population-based study of squamous cell vaginal cancer: HPV and cofactors. Gynecol. Oncol..

[3] Saraiya M (2008). Incidence of in situ and invasive vulvar cancer in the US, 1998–2003. Cancer.

[4] Shah CA, Goff BA, Lowe K, Peters WA, Li CI (2009). Factors affecting risk of mortality in women with vaginal cancer. Obstet. Gynecol...

[5] *National Cancer Institute. Vaginal Cancer Treatment (PDQ)-Health Professional Version*, https://www.ncbi.nlm.nih.gov/books/NBK65801/.

[6] *National Comprehensive Cancer Network. Vulvar Cancer (Squamous Cell Carcinoma) (Version 3.2020)*, https://www.nccn.org/professionals/physician_gls/pdf/vulvar.pdf.

[7] Thigpen JT, Blessing JA, Homesley HD, Berek JS, Creasman WT (1986). Phase II trial of cisplatin in advanced or recurrent cancer of the vagina: A Gynecologic Oncology Group Study. Gynecol. Oncol..

[8] Belinson JL, Stewart JA, Richards AL, McClure M (1985). Bleomycin, vincristine, mitomycin-C, and cisplatin in the management of gynecological squamous cell carcinomas. Gynecol. Oncol..

[9] Tashiro H, Brenner MK (2017). Immunotherapy against cancer-related viruses. Cell Res..

[10] Litwin TR, Clarke MA, Dean M, Wentzensen N (2017). Somatic host cell alterations in HPV carcinogenesis. Viruses.

[11] Le DT (2017). Mismatch repair deficiency predicts response of solid tumors to PD-1 blockade. Science.

[12] Marabelle A (2019). Efficacy of pembrolizumab in patients with noncolorectal high microsatellite instability/mismatch repair-deficient cancer: Results from the phase II KEYNOTE-158 study. J. Clin. Oncol..

[13] Frenel JS (2017). Safety and efficacy of pembrolizumab in advanced, programmed death ligand 1-positive cervical cancer: Results from the phase Ib KEYNOTE-028 trial. J. Clin. Oncol..

[14] Chung HC (2019). Efficacy and safety of pembrolizumab in previously treated advanced cervical cancer: Results from the phase II KEYNOTE-158 study. J. Clin. Oncol..

[15] Naing A (2020). Phase 2 study of pembrolizumab in patients with advanced rare cancers. J. Immunother. Cancer.

[16] Cristescu R (2018). Pan-tumor genomic biomarkers for PD-1 checkpoint blockade-based immunotherapy. Science.

[17] Taube JM (2014). Association of PD-1, PD-1 ligands, and other features of the tumor immune microenvironment with response to anti-PD-1 therapy. Clin. Cancer Res..

[18] Pardoll DM (2012). The blockade of immune checkpoints in cancer immunotherapy. Nat. Rev. Cancer.

[19] Fujii T, Naing A, Rolfo C, Hajjar J (2018). Biomarkers of response to immune checkpoint blockade in cancer treatment. Crit. Rev. Oncol. Hematol..

[20] Fujii T (2018). Incidence of immune-related adverse events and its association with treatment outcomes: The MD Anderson Cancer Center experience. Investig. New Drugs.

[21] Hua C (2016). Association of vitiligo with tumor response in patients with metastatic melanoma treated with pembrolizumab. JAMA Dermatol..

[22] Hosoya K (2020). Association between early immune-related adverse events and clinical outcomes in patients with non-small cell lung cancer treated with immune checkpoint inhibitors. Clin. Lung Cancer.

[23] Reade CJ, Eiriksson LR, Mackay H (2014). Systemic therapy in squamous cell carcinoma of the vulva: Current status and future directions. Gynecol. Oncol..

[24] Naumann RW (2019). Safety and efficacy of nivolumab monotherapy in recurrent or metastatic cervical, vaginal, or vulvar carcinoma: Results from the phase I/II CheckMate 358 trial. J. Clin. Oncol..

[25] Shields LBE, Gordinier ME (2019). Pembrolizumab in recurrent squamous cell carcinoma of the vulva: Case report and review of the literature. Gynecol. Obstet. Investig..

